# Outside and Inside

**Published:** 1907-02

**Authors:** C. A. F. Lindorme

**Affiliations:** Atlanta, Ga.


### OUTSIDE AND INSIDE.

---

By C. A. F. Lindorme, M. D., Atlanta, Ga.

In common parlance we speak of the inside of a body. All right. It does not cause confusion. But in physiological argumentation discrimination is indicated, lest the philosopher be left.

There is in a sense an inside of bodies. It disappears, however, as soon as we approach. Nor do we ever reach it. The alimentary canal is in a measure the inside of an animal body. But it is only so long as it is not brought forward for inspection. The vermiform appendix is an inside, to be sure, very much so, indeed. But in a disease—case, when the surgeon has occasion to cut it out, and lays it on the operating table, it is from an inside turned into an outside. The marrow of the bones is the inner-most organ of the entire anatomy. But in necrosis of a bone the operating surgeon, incidentally to his resection of the same, makes it an outside, and so along the whole line of all the other bodily objects, a church-spire, towering nearly five hundred feet high in the air, and the hold of a gaint ocean steamer, ploughing the main nearly one hundred feet deep, not excluded; everything in the bodily realm is by technical operation, explorative manipulation, or otherwise, from an inside turned to an outside, so that the thesis holds, bodies have an inside so long as it is not made to exhibit outside.

This is clear enough. Nor will there be any sensible philosopher disputing the axiom. But there are certain metaphysical dogmatists who use the word, spirit (or soul or mind), as if soul or spirit or mind had an outside.

It is an old traditional error, which, however, for thousands of years already has forcibly been made a hot controversy.

Honestly, can there be a doubt about what the mind (soul or spirit), is? Can not every doubter be referred to his own spirit (mind or soul?), and does he not get that way plentiful high-grade information?

Or can there be any more reliable information about the soul, (or spirit or mind,) than is afforded by the possession of the same?

One meets sometimes with droll scholars and queer students, men who are foolishly eager to turn things topsy-turvy. In natural science the brave usage is observed to watch a thing in the concrete, and subsequently, according to its peculiarities, make and name a classification in the abstract, and from this laudable custom results steady congruance between the abstract language and the observation of specimens in the concrete.

The metaphysical dogmatists do not follow such a rational course. As mentioned before, they start with a word, some fancied expression, and subsequently hang a tail to it, implying all sorts of reality, of which in reality nothing is in existence.

Supposing, somebody, not quite up-to-date in his personal knowledge ignores what a skirmish is, or a brigade of artillery, and desires information. Well, the best way of doing would be to take him to a manœuvre-camp and let him see both. Or, supposing some other one is ignorant of what a divan is. Well, he is taken to a furniture store and taken over the stock.

Why can the same modus procedenti not be followed in physiology?

We admit, the soul, (or spirit or mind,) cannot be shown, nor observed with the use of our eyesight; it cannot be seen, what you call see in your dictionaries. But can it not be made fully as evident?

What is spirit, (or soul or mind) ?

An answer to this question seems an intricate affair.

It is not in the least. It has been made intricate by creating confusion; that is all. If somebody ignores what the spirit, (or soul or mind) is, he can not be shown it, outside, but he can be made inwardly aware; he can be taken over the stock, for this stock he has in himself. Or is there any individual, worthy of the classification of man, who is so totally without all love, for example, as never to have observed it in himself? And if he should, he is sure to feel hatred, in place of it. Now then, love and hatred are spirit (or mind or soul), so, can the mind, (or soul or spirit,) live, or be taught, nearer-in for ones' information than it actually does? And the same holds with reference to all other mind, (or soul or spirit). Courage and cowardice, intrepidity, and pusillanimity, foolhardiness and weariness, gentleness and rudeness, lavishness and stinginess, pleasantness and surliness, hotheadedness and sluggishness, etc. etc., all these conditions are specimens of spirit, (or soul or mind,) and affirm our theory, that there is no outside to the spirit, (or soul or mind) : on the one hand bodies have no inside, on the other hand, mind (or soul or spirit,) has no outside; it might for the matter of that be called an absolute inside, or, better, inwardness.

This settles the point regarding mind, (or soul or spirit,) it is our emotions, our fundamental drift, to use an old, much too much neglected excellent term, the drift from which is born our will, our passions and all that sort of thing, and brought on a general expression, a wider abstraction. By this bringing of the related conditions on a general expression, the subject matter, however, has by no manner of means been made more intricate; love and generosity, by being called spirit, are fully as plain and homely as before. Same thing with other emotions; by using the terminology we do not alter anything in the argument; all we do alter is the order of the abstract classification; we make a class of a wider scope of thought, a class which summarizes antagonistic emotions over the head of other groups; cowardice in this summarizing marches up with courage, for cowardice is as well spirit, (or mind or soul,) as courage, although of a rather disreputable parentage, in how much it must remain for the concrete case to find out; it does not effect the mere spirit question which refers to the bodily contrast alone.

There are fanciful scholars who are hot after definitions, definitions at any cost, as if it was the definitions which embrace all the wisdom. Go, then, for a definition, or rather two, one for the bodies another for the soul (or mind or spirit).

Strictly speaking, one should suppose that a definition of bodies was unnecessary. It is notorious that a body is an object to the perception of which the function of the nerves of special sense are subservient. But since it is not at all a rarety, that, as stated as above, things are perfectly plain in the concrete, which, being brought on a more or less general expression, become doubtful and are called into question, we will be more explicit, and say, that bodies are such objects as, one way or another, have a bearing upon our own body. So long as such a bearing has not been observed and demonstrated, the existence of the claimed body has not been made evident; and the peculiarities of the body in question depend always on the bearing of the same on our own body. That is the reason why foreign bodies, and our own body, indeed, no less, is ultimately ever an outside affair; in order to make sure of the existence of a body, we have to see, to hear, to smell, to taste, or to touch it.

Perfectly plain, but not exactly new, the reader will mutter.

Nor would there be occasion for such homely truths to utter, if the full use of the five senses was not so altogether uncommon, and pertinent mother-wit at a premium.

Now, for a definition of the mind (or spirit or soul), which, delivered from the height of our preliminary analytical research, the reader will not be surprised to find somewhat peculiar. It is original, to be sure, I, at least, encountered its like nowhere.

But its truth is none the less indisputable.

Before mentioning, however, the definition itself, we will state the contrasting mental corollary of the definition of bodies. Mental impressions, or subjects, are such as have no bearing upon our own body, a bearing upon our soul, (mind or spirit) alone. Already some centuries ago a French psychologue gave us the aphorism, it is the spirit alone which feels the spirit; (ce n'est que l'esprit qui sente l'esprit) and correspondly only modalities will be recognized.

The definition which we derive from the investigation of the facts, which we let go before is, the mind, (or soul, or spirit,) is the intimacy of our, of all being in the world.

Nor is this definition a truism. It is on the contrary of the utmost consequence. We are compelled to draw the amazing inference, that all detail of observation and consequently all difference of structure is restricted to the bodily world, to corporeal objects It is absolutely impossible to give a description of any detail of difference of the spirit, (or soul or mind;) the soul, (or mind or spirit,) as the intimacy of our, of all being in the world, is, wherever met, and ever-everywhere a totality.

Bodies separate; the mind, (or soul or spirit,) unites.

Our statements may be startling to the metaphysical dogmatist and the timid empiric. We admit, it must be amazing at first sight to the rupted physchologue and the physchological novice, must be, but is sure to work beneficially when put practically to an actual test. At any rate there is no other scientific foundation of morality, of really civilized society, and on account of its overwhelming significance the reader will kindly allow us, to spin out the axiom to the full length and breadth of the scope of its enunciation:

As to soul-life, (mentally or spiritually,) there is no difference from man to man.

As to soul-life, (mentally or spiritually,) there is no difference from man to animals.

As to soul-life, (mentally or spiritually,) there is no difference from man to trees.

As to soul-life, (mentally or spiritually,) there is no difference from man to flowers, grass or shrubberies.

As to soul-life, (mentally or spiritually,) there is no difference from man to air or gas.

As to soul-life, (mentally or spiritually,) there is no difference from man to floods or oceans.

As to soul-life, (mentally or spiritually,) there is no difference from man to rivers, springs or brooks.

As to soul-life, (mentally or spiritually,) there is no difference from man to rock.

As to soul-life, (mentally or spiritually,) there is no difference from man to clay.

There is difference. But it is altogether in the bodily proportions. If the mind works differently, it is on account of the bodily instrumentality; mental inferiority cannot be thought irrespective of defective bodily evolution, nor can superiority occur without corresponding bodily perfection.

Hence the immense importance of the bodily detail, and the extent of its significance in breeding and education, as yet not by far sufficiently recognized and appreciated.

A very useful example is energy. No sensible psychologue will dispute the spirituality of energy, (or its mentality, its soul-quality,) but neither will a sensible psychologue dispute the necessity of bodily perfection for the energy to work through. It is very characteristic that Marconi designated his invention, wireless telegraphy. He left it an open question how the power had to be comprehended otherwise, but maintained in his terminology, that it could not be understood independently from corporeality.

This implies, however, inversely, that all bodily being is endowed with soul, (or mind or spirit).

Furthermore we will state, to a hoped-for satisfaction of the appallingly large number of those who have unspeakable trouble to break themselves from the uncanny habit inveterate as old, of treating mind, (or soul or spirit,) as a multiple, shaped and subject to measurement, that there is a scientific point of difference in spirit, (mind or soul,) which we have to concede, but do not either hesitate to do it, for the difference has reference only to the degree to which mind, (or soul or spirit,) is effectual in its work through the bodies.

Finally an empirical point may be mentioned which seems to militate against our theory, but does it in reality so little, that, on the contrary, it endorses our plan. It is the promiscuous employment of the identical attributes, hither and thither in the dictionaries, in the mental field and in the bodily realm. The indifferent usage of the same terminology, hither and thither, is not unconditional; it is mere accommodation; the dictionaries themselves warn against an abuse; the indifferent usage in the bodily field, and in the mental domain is justified with the restriction only, that, with reference to the application to mental, (or spiritual or soul-life objects,) the sense is only metaphorical.

